# 马立巴韦治疗3例异基因造血干细胞移植后HHV-6B感染的疗效及安全性

**DOI:** 10.3760/cma.j.cn121090-20250206-00054

**Published:** 2025-07

**Authors:** 承伟 金, 肃 李, 璐祥 王, 佳瑜 黄, 晓霞 胡, 子璐 张

**Affiliations:** 1 上海交通大学医学院附属瑞金医院血液科，上海血液学研究所，国家转化医学研究中心，上海 200025 National Research Center for Translational Medicine, State Key Laboratory of Medical Genomics, Shanghai Institute of Hematology, Ruijin Hospital, Shanghai JiaoTong University School of Medicine, Shanghai 200025, China; 2 上海力泉医院血液科，上海 201418 Department of Hematology, Shanghai Liquan Hospital, Shanghai 201418, China

## Abstract

异基因造血干细胞移植后人类疱疹病毒-6B型（HHV-6B）感染是移植后非复发死亡的重要原因之一。回顾性分析3例异基因造血干细胞移植后HHV-6B再激活患者，以膦甲酸钠为一线抗病毒方案无效/不耐受，马立巴韦治疗后病毒转阴。1例HHV-6B脑炎患者无神经系统后遗症，3例患者均未观察到药物不良反应，初步证实马立巴韦可用于治疗移植后常规治疗无效/不耐受的HHV-6B感染。

## Abstract (en)

Human herpesvirus-6B（HHV-6B）reactivation is a significant contributor to nonrelapse mortality following allogeneic hematopoietic stem cell transplantation（allo-HSCT）. This retrospective analysis describes three cases of HHV-6B reactivation following allo-HSCT, all of which achieved viral clearance with maribavir treatment following failure or intolerance to first-line antiviral therapy with foscarnet sodium. One patient diagnosed with HHV-6B encephalitis recovered without neurological sequelae. No adverse drug reactions to maribavir were observed. These findings provide preliminary evidence that maribavir may serve as an effective and safe salvage therapy for HHV-6B reactivation in patients receiving allo-HSCT.

异基因造血干细胞移植（allo-HSCT）是多种血液系统疾病的治愈手段，但免疫抑制增加病毒再激活风险，是移植后非复发死亡的重要原因之一[1]–[3]。人类疱疹病毒6型（HHV-6）属于人类疱疹病毒科，包括HHV-6A和HHV-6B两种亚型：HHV-6A亚型基因组伴有广泛的组蛋白修饰，处于转录沉默状态；HHV-6B通常在人类6个月至2岁感染且建立终生潜伏，在免疫抑制时再激活，是移植患者感染的主要病毒类型之一[4]–[5]。

HHV-6B再激活诊断依赖PCR检测血液和（或）组织标本HHV-6B DNA[6]，近年来宏基因组二代测序（mNGS）进一步提高检测灵敏度[7]。HHV-6B再激活通常发生在移植后早期，中位激活时间21 d，病毒载量快速升高，从病毒检出至终末器官疾病时间间隔短（中位时间5 d）[8]。HHV-6B再激活与植入延迟、植入失败、急性移植物抗宿主病（aGVHD）、移植相关血栓性微血管病变（TA-TMA）、病毒性脑炎和肺炎相关，导致非复发死亡率增高[9]–[11]。欧洲白血病感染会议（ECIL）造血干细胞移植后感染管理指南推荐HHV-6再激活一线治疗为更昔洛韦、膦甲酸钠，二线方案为西多福韦，但药物的脏器毒性及骨髓抑制效应限制了其临床应用及疗效。我们回顾性分析3例马立巴韦成功治疗allo-HSCT后HHV-6B感染且更昔洛韦/膦甲酸钠治疗无效的病例资料并进行文献复习，现报道如下。

## 病例资料

例1，男，52岁，诊断为骨髓增生异常综合征［分子国际预后评分系统（IPSS-M）极高危，修订的国际预后评分系统（IPSS-R）极高危］，维奈克拉联合阿扎胞苷诱导缓解后进行allo-HSCT。+13 d出现高热，最高体温（*T*_max_）40.0 °C；血常规：WBC 0.3×10^9^/L，HGB 62 g/L，PLT 21×10^9^/L；外周血HHV6-DNA（PCR法）：4.79×10^4^拷贝/ml，外周血mNGS：HHV-6B序列数197条。诊断：HHV-6B病毒血症。予下调钙调磷酸酶抑制剂（CNI）浓度，加用膦甲酸钠180 mg·kg^−1^·d^−1^抗病毒、辅以丙种球蛋白（0.4 g·kg^−1^·d^−1^，3 d）治疗。+16 d出现听力下降及记忆力减退，外周血HHV6-DNA：1.82×10^5^拷贝/ml。脑脊液压力：218 mmH_2_O；脑脊液生化：总蛋白1 144.4 mg/L，葡萄糖3.14 mmol/L，氯化物121.9 mmol/L，乳酸脱氢酶57.7 U/L；脑脊液常规：透明度清，WBC 10×10^6^/L，RBC 6.5×10^9^/L，潘氏蛋白实验阳性；脑脊液HHV6-DNA（PCR法）：<1×10^3^拷贝/ml，脑脊液mNGS：HHV-6B序列数1 002条。加用更昔洛韦5 mg·kg^−1^·d^−1^联合治疗。+23 d外周血HHV6-DNA：6.7×10^5^拷贝/ml，脑脊液HHV6-DNA（PCR法）：1.6×10^3^拷贝/ml，脑脊液mNGS：HHV-6B序列数1 238条。调整抗病毒方案为马立巴韦0.4 g每日2次。+32 d外周血和脑脊液HHV-6 DNA均转阴，马立巴韦抗病毒2周后停药，无中枢神经系统后遗症。

例2，男，61岁，诊断急性髓系白血病（AML），第1次完全缓解期进行allo-HSCT。+18 d出现高热，*T*_max_ 39.4 °C；血常规：WBC 0.53×10^9^/L，HGB 52 g/L，PLT 16×10^9^/L；外周血HHV6-DNA（PCR法）：1.26×10^4^拷贝/ml，外周血mNGS：HHV-6B序列数171条。诊断：HHV-6B病毒血症。予下调CNI浓度，加用膦甲酸钠180 mg·kg^−1^·d^−1^抗病毒治疗。+25 d复查外周血HHV6-DNA：3.04×10^5^拷贝/ml，肌酐190 µmol/L，肌酐清除率0.5 ml·min^−1^·kg^−1^。提示膦甲酸钠不耐受，调整抗病毒方案为马立巴韦0.4 g每日2次。+35 d外周血HHV-6 DNA转阴。

例3，女，61岁，诊断AML，第1次完全缓解期进行allo-HSCT。+13 d粒系和血小板植入，未发生aGVHD。移植后8个月出现发热，*T*_max_ 38.7 °C；血常规：WBC 1.56×10^9^/L，中性粒细胞计数1.38×10^9^/L，HGB 41 g/L，PLT 32×10^9^/L；外周血CD4^+^细胞绝对计数：421个/µl；外周血HHV6-DNA（PCR法）：1.50×10^4^拷贝/ml，外周血mNGS：HHV-6B序列数934条，脑脊液mNGS阴性。诊断：HHV-6B病毒血症。给予膦甲酸钠120 mg·kg^−1^·d^−1^抗病毒、辅以丙种球蛋白（0.4 g·kg^−1^·d^−1^，3 d）治疗，7 d后复查外周血HHV6-DNA 7.70×10^4^拷贝/ml，合并2级静脉炎及顽固性低钾血症。调整抗病毒方案为更昔洛韦5 mg·kg^−1^·d^−1^，1周后复查外周血HHV6-DNA 9.6×10^4^拷贝/ml，期间反复发热，调整抗感染方案为马立巴韦0.4 g每日2次，7 d后复查外周血HHV-6 DNA转阴。

## 讨论

HHV-6B再激活多发生于allo-HSCT后早期[12]。更昔洛韦和膦甲酸钠是HHV-6B再激活的一线治疗方案[9]，西多福韦为二线方案[13]。由于膦甲酸钠能透过血脑屏障，神经系统症状缓解率83.8％，日本造血干细胞移植学会指导委员会推荐膦甲酸钠为HHV-6B脑炎一线治疗[14]，更昔洛韦和膦甲酸钠联合用药用于严重感染[15]，但药物不良反应往往限制其足剂量足疗程使用。

免疫缺陷人群中，长程及亚标准剂量使用抗病毒药物与耐药相关。疱疹病毒耐药突变大多发生于胸苷激酶（TK）、蛋白激酶（PK），其次为DNA聚合酶（DNApol）[16]。HHV-6B编码PK的U69基因突变及编码DNApol的U38基因突变改变蛋白催化结构域从而导致更昔洛韦耐药[17]–[19]，且存在更昔洛韦及西多福韦交叉耐药株[20]，是移植后非复发死亡的重要原因之一。鉴于耐药株及抗病毒药物的不良反应，allo-HSCT后免疫缺陷患者HHV-6B感染、特别是HHV-6B终末器官疾病（如肺炎、脑炎）亟待新的治疗策略。

马立巴韦是新型苯并咪唑核糖苷类药物，竞争性抑制巨细胞病毒（CMV）pUL97蛋白激酶活性，抑制病毒DNA复制、包壳及核输出[21]，一项多中心、随机、开放标签的Ⅲ期临床研究纳入352例干细胞移植后难治性CMV感染患者，与传统抗病毒治疗相比，马立巴韦显著提高病毒转阴率（23.9％对55.7％，*P*<0.001）[21]，已获批用于治疗更昔洛韦、缬更昔洛韦、西多福韦或膦甲酸钠难治的移植后CMV感染[22]–[23]。马立巴韦有更低的脏器损伤和骨髓抑制效应，病毒清除率高，中位清除时间短[24]。HHV-6与CMV均属于β-疱疹病毒，其核苷酸高度同源[25]，体外实验及临床疗效证实HHV-6与CMV抗病毒治疗谱类似[16]。pUL97蛋白激酶在疱疹病毒家族成员中高度保守，pU69为其在HHV-6中的同源类似物[26]，参与病毒复制过程中细胞周期蛋白磷酸化及再分布[17]。体外实验证实马立巴韦可有效抑制U69激酶活性[27]。目前尚无研究报道马立巴韦在HHV-6B感染中的疗效。我们回顾性分析了马立巴韦治疗3例移植后HHV-6B再激活病例，均获得了病毒持续转阴。马立巴韦治疗期间未出现药物相关骨髓抑制和肾脏毒性等不良反应。初步证实马立巴韦在移植后HHV-6B感染中的疗效，为更昔洛韦/膦甲酸钠不耐受或耐药的HHV-6感染提供新的治疗选择。例1中马立巴韦有效降低外周血和脑脊液HHV-6B病毒载量，为马立巴韦中枢神经系统渗透提供了有力佐证。但本研究样本量较少、随访时间较短，未来应开展前瞻性临床研究纳入更大样本量验证。

## References

[1] 中华医学会血液学分会干细胞应用学组 (2022). 异基因造血干细胞移植患者巨细胞病毒感染管理中国专家共识(2022年版)[J]. 中华血液学杂志.

[2] 李 瑶, 张 枫, 刘 畅 (2024). 成人血液病患者异基因造血干细胞移植后呼吸道合胞病毒感染的临床特征及死亡相关危险因素分析[J]. 中华血液学杂志.

[3] 中华医学会血液学分会, 中国医师协会血液科医师分会 (2022). 造血干细胞移植后EB病毒相关淋巴增殖性疾病中国专家共识(2022年版)[J]. 中华血液学杂志.

[4] Agut H, Bonnafous P, Gautheret-Dejean A (2015). Laboratory and clinical aspects of human herpesvirus 6 infections[J]. Clin Microbiol Rev.

[5] Tesini BL, Epstein LG, Caserta MT (2014). Clinical impact of primary infection with roseoloviruses[J]. Curr Opin Virol.

[6] Ward KN, Hill JA, Hubacek P (2019). Guidelines from the 2017 European Conference on Infections in Leukaemia for management of HHV-6 infection in patients with hematologic malignancies and after hematopoietic stem cell transplantation[J]. Haematologica.

[7] Carpenter ML, Tan SK, Watson T (2019). Metagenomic Next-Generation Sequencing for Identification and Quantitation of Transplant-Related DNA Viruses[J]. J Clin Microbiol.

[8] Hill JA, Mayer BT, Xie H (2018). Kinetics of Double-Stranded DNA Viremia After Allogeneic Hematopoietic Cell Transplantation[J]. Clin Infect Dis.

[9] Quintela A, Escuret V, Roux S (2016). HHV-6 infection after allogeneic hematopoietic stem cell transplantation: From chromosomal integration to viral co-infections and T-cell reconstitution patterns[J]. J Infect.

[10] Yu Y, Chen W, Fu H (2024). Risk factors and long-term outcomes for human herpesvirus 6 encephalitis in the early period after allogeneic stem cell transplantation[J]. Bone Marrow Transplant.

[11] Hill JA, Lee YJ, Vande Vusse LK (2024). HHV-6B detection and host gene expression implicate HHV-6B as pulmonary pathogen after hematopoietic cell transplant[J]. Nat Commun.

[12] 黄 莉, 韩 婷婷, 魏 芳芳 (2024). 异基因造血干细胞移植后HHV-6病毒感染的临床特征分析[J]. 中华血液学杂志.

[13] Pöhlmann C, Schetelig J, Reuner U (2007). Cidofovir and foscarnet for treatment of human herpesvirus 6 encephalitis in a neutropenic stem cell transplant recipient[J]. Clin Infect Dis.

[14] Irie K, Hiramoto N, Yamaoka K (2023). Cerebrospinal fluid concentration of foscarnet in patients with HHV-6 encephalitis after haematopoietic stem cell transplantation[J]. J Antimicrob Chemother.

[15] Ogata M, Uchida N, Fukuda T (2020). Clinical practice recommendations for the diagnosis and management of human herpesvirus-6B encephalitis after allogeneic hematopoietic stem cell transplantation: the Japan Society for Hematopoietic Cell Transplantation[J]. Bone Marrow Transplant.

[16] Topalis D, Gillemot S, Snoeck R (2018). Thymidine kinase and protein kinase in drug-resistant herpesviruses: Heads of a Lernaean Hydra[J]. Drug Resist Updat.

[17] Bonnafous P, Boutolleau D, Naesens L (2008). Characterization of a cidofovir-resistant HHV-6 mutant obtained by in vitro selection[J]. Antiviral Res.

[18] Piret J, Boivin G (2014). Antiviral drug resistance in herpesviruses other than cytomegalovirus[J]. Rev Med Virol.

[19] Topalis D, Gillemot S, Snoeck R (2016). Distribution and effects of amino acid changes in drug-resistant α and β herpesviruses DNA polymerase[J]. Nucleic Acids Res.

[20] Manichanh C, Olivier-Aubron C, Lagarde JP (2001). Selection of the same mutation in the U69 protein kinase gene of human herpesvirus-6 after prolonged exposure to ganciclovir in vitro and in vivo[J]. J Gen Virol.

[21] Avery RK, Alain S, Alexander BD (2022). Maribavir for Refractory Cytomegalovirus Infections With or Without Resistance Post-Transplant: Results From a Phase 3 Randomized Clinical Trial[J]. Clin Infect Dis.

[22] Kang C (2022). Maribavir: First Approval[J]. Drugs.

[23] Mullard A (2022). FDA approves decades-old maribavir for CMV infection[J]. Nat Rev Drug Discov.

[24] 马 薇, 魏 志杰, 卢 岳 (2024). 马立巴韦治疗异基因造血干细胞移植后难治与药物不耐受CMV血症和CMV病25例临床分析[J]. 中华血液学杂志.

[25] Prichard MN, Frederick SL, Daily S (2011). Benzimidazole analogs inhibit human herpesvirus 6[J]. Antimicrob Agents Chemother.

[26] Michel D, Mertens T (2004). The UL97 protein kinase of human cytomegalovirus and homologues in other herpesviruses: impact on virus and host[J]. Biochim Biophys Acta.

[27] Prichard MN, Frederick SL, Daily S (2011). Benzimidazole analogs inhibit human herpesvirus 6[J]. Antimicrob Agents Chemother.

